# Dose-response relationship of high-intensity training on global cognition in older adults with mild cognitive impairment or dementia: a systematic review with meta-analysis - the ACHIEVE-Study

**DOI:** 10.1186/s11556-024-00358-3

**Published:** 2024-09-16

**Authors:** Diego Fernando Afanador-Restrepo, Alejandro Casanova-Correa, Rita Inés Martín-Ojeda, Agustín Aibar-Almazán, Ana María González-Martín, Fidel Hita-Contreras, María del Carmen Carcelén-Fraile, Yolanda Castellote-Caballero

**Affiliations:** 1 Faculty of Health Sciences and Sport, University Foundation of the Área Andina, Pereira, Colombia; 2 Santa Cruz Rehabilitation Center, Las Palmas de Gran Canaria, 35011 Spain; 3 Escaleritas Rehabilitation Center, Las Palmas de Gran Canaria, 38001 Spain; 4 Department of Health Sciences, Faculty of Health Sciences, University of Jaén, Jaén, 23071 Spain; 5 Department of Education and Psychology, Faculty of Social Sciences, University of Atlántico Medio, Las Palmas de Gran Canaria, 35017 Spain; 6 Department of Health Sciences, Faculty of Health Sciences, University of Atlántico Medio, Las Palmas de Gran Canaria, 35017 Spain

**Keywords:** High-intesity training, Alzheimer’s disease, Aging, Mild cognitive impairment

## Abstract

**Background:**

The prevalence of mild cognitive impairment (MCI) and its subsequent progression to dementia has increased progression to dementia has increased worldwide, making it a topic of interest. of interest, and it has been observed that approximately 23% of cases are avoidable through preventable through vigorous exercise.

**Methods:**

A systematic review with meta-analysis was conducted by searching in the PubMed, Scopus, CINAHL, and Web of Science databases. For inclusion, studies had to incorporate High Intensity Training (HIT) as a primary or significant component of the overall intervention for older adults with MCI. Out of the 611 articles identified, 14 randomized clinical trials met the criteria for inclusion in the review.

**Results:**

Fourteen trials were included in the systematic review, and seven were included in the meta-analysis. A total of 1839 participants were included in the studies, with 1014 receiving a high-intensity training-based intervention, and 998 were considered in the meta-analysis. Compared to usual care or sedentary activities, the high-intensity training interventions had a positive effect on cognition, either improving it or delaying the decline (*g* = 0.710 (95% CI: 0.191 — 1.229; *p* = 0.007). Additionally, the meta-analysis determined that a frequency of 3 sessions per week (g = 0.964, CI = 0.091 — 1.837, *p* = 0.030) of approximately 60 minutes (g = 0.756, CI = 0.052 — 1.460, *p* = 0.035) each was the best dose to obtain better effects on global cognition.

**Conclusion:**

Low-frequency and short-duration high-intensity training interventions are sufficient to improve or at least delay the decline in global cognition.

## Introduction

 In Colombia, as in much of the world, a demographic transition has been taking place for many years, as evidenced by an inverted population pyramid [1]. Currently, it is possible to observe an increase in the older adult population, which can be attributed to improvements in the quality of life. Conversely, the younger population has experienced a decline as a result of decreasing birth rates [2]. The changes in population dynamics pose a problem that was not considered in previous decades, namely the burden on the healthcare system arising from aging and its detrimental effects on people’s health [3]. According to data published by the National Administrative Department of Statistics (DANE) of Colombia, the aging index has quadrupled between 1950 and 2020. In 1950, there were 12 older adults for every 100 children under 15 years of age, whereas in 2020, the number of older adults increased to 49 [4].

Current literature indicates that aging has a significant negative impact on various areas of the brain, including the prefrontal, temporal, and hippocampal regions. It also leads to a decrease in brain volume and blood flow, which results in memory and cognitive impairments [5–9]. Consequently, a decline in cognitive functions is expected as individuals age [10]. In this context, mild cognitive impairment (MCI) emerges as a critical stage in the aging process. MCI is defined as a decline in cognitive abilities that does not meet the threshold for dementia. However, research suggests that MCI may serve as a precursor to dementia, particularly Alzheimer’s disease [11].

It is important to highlight that MCI affects not only a small group of individuals but also poses a significant public health challenge worldwide. The prevalence of MCI and its progression to dementia in the older adult population are substantial and increasing, with an estimated 78 million cases expected by 2030 [12]. Therefore, effective strategies are needed to prevent the progression of MCI and alleviate its impact on the quality of life of older adults [13].

In this regard, scientific research has demonstrated the crucial role that physical exercise and other interventions can play in maintaining and improving cognitive function in the older adult population [14–16]. Furthermore, this issue has garnered the attention of various researchers, highlighting the necessity to explore strategies that can halt its natural progression toward dementia and its potentially fatal outcome [17].

Regarding physical exercise, one of the option used is High-intensity training (HIT). This type of training is typically characterized by exerting an effort that exceeds 60–80% of one’s maximum repetition (1RM) during resistance or strength training [18]. In the case of cardiopulmonary exercise, it involves maintaining a target intensity ranging from 80 to 100% of the maximum heart rate (HRmax) [19]. This type of exercise has demonstrated a positive impact on various physical and cognitive variables. It has been shown to improve factors such as fall risk, balance, gait, independence, memory, verbal fluency, attention, and global cognition [16, 20–23]. Moreover, HIT has been found to influence cognitive improvement in older adults with MCI or dementia by enhancing blood flow and promoting neuroplasticity [24]. Additionally, the exercise in general can enhance cardiovascular function and reduce the risk of chronic degenerative diseases. These benefits can also have a positive impact on cognitive health from a neurobiological perspective [25].

Different authors have demonstrated, through systematic reviews, that the heterogeneity observed among the different studies makes it impossible to attribute the effects to a standardized exercise protocol [26, 27]. However, evidence suggests HIT is the most effective type of exercise for mitigating the effects of aging on cognition by increasing the production of brain-derived neurotrophic factor (BDNF, at least in young adults [28]. Additionally, Nascimento et al. [29] observed that aerobic exercise reaching 80% of the maximum heart rate, estimated by Karvonen’s formula, was able to improve cognitive function in cognitively impaired older adults through the increase of BDNF. Therefore, the objectives of the current systematic review with meta-analysis were to determine the optimal dose-response relationship of HIT to achieve maximum therapeutic effects in improving cognition in older adults with MCI or dementia.

Through this research, we aim to make a valuable contribution to the development of physical exercise-based interventions that can effectively delay the progression of MCI or dementia and enhance the quality of life for older adults. Moreover, we anticipate that our study will provide robust scientific evidence to support the implementation of personalized and effective exercise strategies in this expanding population group.

## Methodology

This systematic review with meta-analysis aims to determine the optimal dose-response relationship of HIT for achieving maximum therapeutic effects in improving cognition among older adults with MCI or dementia. The development of this review adhered to the guidelines outlined in the PRISMA 2020 document [30] and followed the procedures described in the Cochrane Manual for the Elaboration of Systematic Reviews of Interventions [31]. The review protocol was registered and pre-specified in PROSPERO under the code CRD42023408275.

### Eligibility criteria

#### Inclusion criteria

The articles included in this review had to meet the following inclusion criteria: (i) Studies that utilized HIT as part of the treatment for older adults with MCI or dementia in the experimental group; (ii) Randomized clinical trials; (iii) Objective measurement of cognition before and after the exercise intervention. These criteria were employed to ensure that the selected studies focused on HIT interventions, employed rigorous experimental designs, and used objective measures to assess changes in cognition before and after the exercise intervention.

#### Exclusion criteria

The following exclusion criteria were applied in this systematic review with meta-analysis: studies that did not measure the relevant study variables; to ensure the extrapolation of the results, studies focused on ethnic minorities, populations with limited mobility, history of psychiatric disorders, acute infections, neurological diseases, and hormonal disorders were excluded. Additionally, studies that lacked an acceptable level of internal and external validity (PEDro scale < 6) were excluded. Moreover, publications such as books, meta-analyses, reviews, systematic reviews, protocols, clinical trial registries, and non-peer-reviewed articles were discarded from the analysis. These criteria ensured that the selected studies had a sufficient level of methodological rigor and relevance for the review.

### Information sources

A literature search was conducted between October and December 2023 in Pubmed, Scopus, Web of Science and CINAHL databases.

### Search strategy

Keywords were used through the following search strategy: (“high-intensity training” OR “high-intensity exercise” OR “HIIT” OR “High-Intensity exercise training” OR “high intensity training” OR “high intensity exercise” OR “HIT” OR “High Intensity exercise training” OR “resistance training” OR “Physical exercise” OR “intensive training program” OR “circuit training” OR “dance” OR “high-intensity”) AND (“older adults with cognitive impairment” OR “cognitive impairment” OR “Cognitive decline” OR “Dementia”) AND (“global cognition” OR “Cognition” OR “cognitive function”).

### Selection process

The search results obtained were processed using the Rayyan QCRI application [32] (https://rayyan.qcri.org/welcome) which automatically eliminated duplicate articles. Two authors (D.F.A.-R and A.C.-C.) independently and blindly reviewed the titles and abstracts of the remaining articles to assess their compliance with the inclusion criteria. Subsequently, they read the full-text articles. Any discrepancies or disagreements in the selection process were resolved through consensus with a third author (A.A.-A.).

### Data collection extraction

The main variables of this review focused on measuring outcomes related to cognition. Each included article was classified based on its year of publication, country, author/s, characteristics of the participants (age, sample size and group distribution), intervention to be followed by the experimental and control groups (duration of the intervention, duration of each session and frequency as well as the intensity measure), type of variable, test used and follow-up time.

### Methodological quality assessment

The methodological quality of the selected articles in this review was assessed using the PEDro scale, which is a widely utilized scale for evaluating methodological quality. The scores were obtained from the PEDro website whenever available. If not available, two authors (D.F.A.-R. and M.R.M.-B.) independently evaluated the studies. In case of any discrepancies, a third author (A.M.G.-M) resolved them. The PEDro scale consists of eleven items that assess the internal and external validity as well as statistical support of the publication [33]. The first item, which pertains to external validity, is not included in the final score calculation. Each of the remaining items was scored as either one (if the criterion is met) or zero (if the criterion is not met) in the publication. The sum of the scores for the second to eleventh items determined the overall score, which ranged from zero to ten points. The overall scores were categorized as follows: 0–3 points (poor quality), 4–5 points (fair), 6–8 points (good), and > 9 points (excellent).

### Effect measures

A meta-analysis was conducted to consolidate the dose-response relationship of the HIT intervention in the older adult population. Furthermore, an outlier analysis was performed within the meta-analysis to identify and assess the influence of any potential outliers on the pooled results. To achieve this, a sensitivity analysis was conducted by excluding the identified outliers and comparing the results with those obtained in the original analysis. Any differences between the sensitivity analysis and the original analysis were carefully examined, and this process ensured the comprehensive inclusion of all relevant articles while maintaining the integrity of the findings.

The selection between the random effects model and the fixed effects model will depend on the level of heterogeneity and variability identified through Cochrane Q and I^2^ statistics. Forest plots were used to visually present the results of the meta-analysis. The forest plots included information such as the name of the first author, the publication date, individual effects (reported as Hedge’s g or Difference in means), the overall effect with its 95% confidence interval (CI), and the associated p-value for each statistic.

Subgroup analysis or stratified analysis was conducted by grouping studies based on the frequency, duration, and volume of interventions. This analysis aimed to examine the effect size and variability within each subgroup, providing a more comprehensive and detailed understanding of the results. Additionally, the risk of publication bias was assessed using a funnel plot.

## Results

### Study selection

**Fig. 1:**
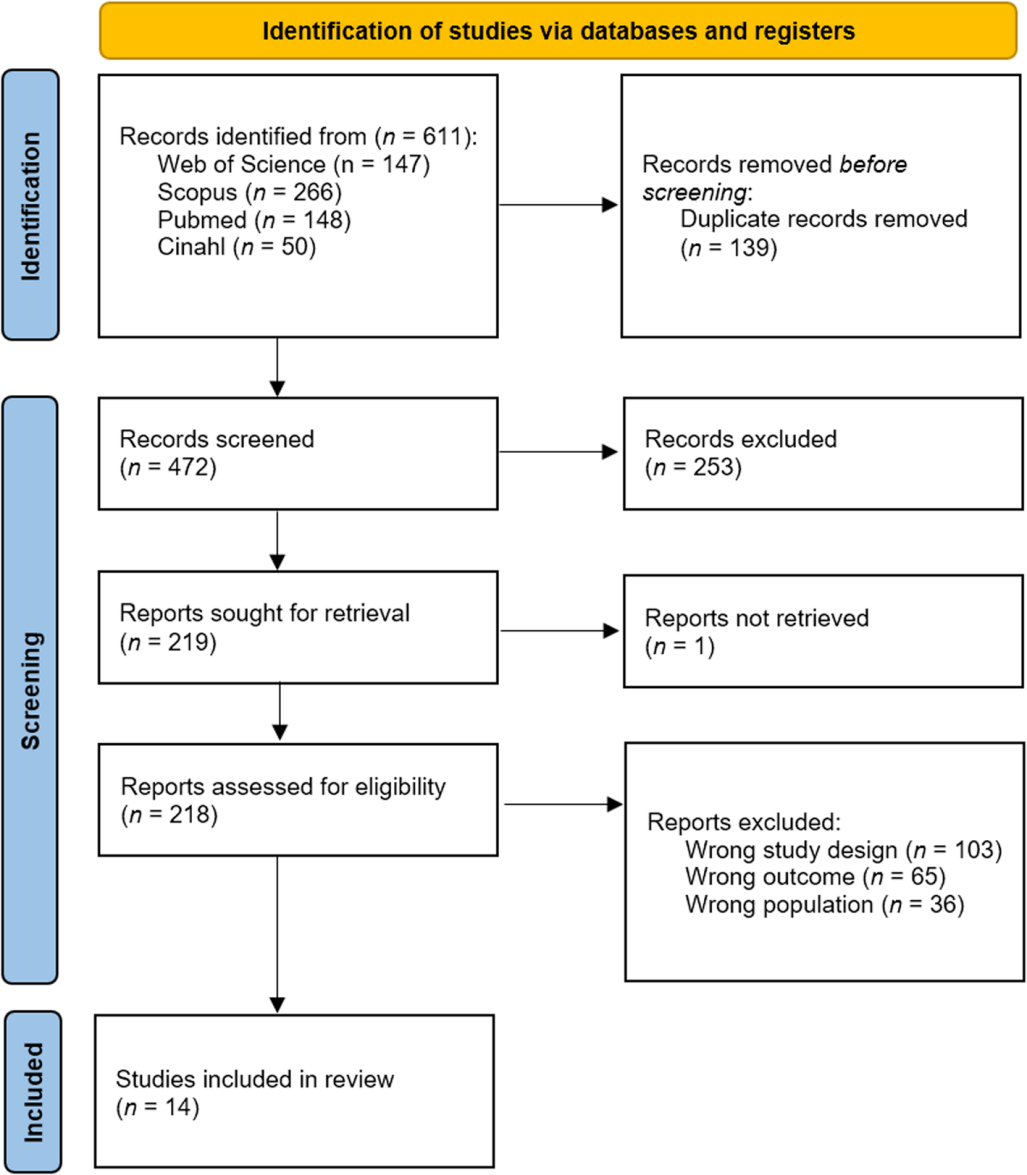
PRISMA Flow diagram of the study selection

A comprehensive search was conducted across various databases, resulting in a total of 611 articles. Prior to the screening process, 139 duplicate articles were eliminated, leaving a total of 472 distinct articles. These articles were then screened based on their titles and abstracts, resulting in 219 articles that were further reviewed in full text. Out of these, 14 articles [34–47] were included in the systematic review, while 204 articles were excluded. The study selection process, following the PRISMA statement [48], is illustrated in Fig. 1.

### Study characteristics

All of the articles included in this systematic review with meta-analysis were randomized controlled clinical trials. The studies were published in various countries including United States [34–36, 39, 40, 43–45], Netherlands [37, 46], Switzerland [42], New Zealand [47], United Kingdom [38] and the United Arab Emirates [41]. However, it is important to note that while the articles were published in these countries, the actual research was conducted in different locations such as Netherlands [34, 43], Norway [44, 45], Colombia [42], Australia [35], Brazil [41], Canada [39], China [47], Denmark [37], Italy [40], Nigeria [36], United Kingdom [38] and Sweden [46]. The studies were conducted between 2014 and 2023, with 2015 being the year with the highest number of publications [34, 37, 44, 45].

A total of 1839 individuals participated in the studies included in this review. Among them, 825 participants were assigned to the control group who received usual care (*n =* 431) or low-intensity exercise (*n* = 394), while 1014 received a HIT intervention. The mean age of the participants across the studies was 77.85 ± 6.64 year (Tables 1 and 2). Among the included studies, only one [47] directly measured the HRmax, while the others relied on estimation and subjective control methods, such as using formulas to calculate HRmax or assessing perceived exertion rate.

**Table 1:** Effects of High-Intensity Training on global cognition in older adults with mild cognitive impairment or dementia

Author and Year	Dementia/ MCI	SampleCG/IG	Control Group	Intervention Group				
				Age	Intervention type	Assessments times	Modification of the outcome over time	Study results
Bossers et al., 2015 [34]	Dementia	CG= 36IG= 37	one-on-one social visits	CG 85.4±5.0IG 85.7±5.1	Strength and AerobicTrainingI: 12 - 15 borg; 50 - 85%HRmaxF: 4 days/weekD: 30 minutes#S: 36	T0=Baseline T1= 9 weeks	MMSE T0= 15.8±4.3T1= 17.16±4.33	Compared to a non-exercise control group, a combination of aerobic and strength training is more effective than aerobic-only training in slowing cognitive and motor decline in patients with dementia.
Fiatarone et al., 2014 [35]	MCI	CG= 27IG= 27	SCOG and SPEX	55–89	Progressive Resistance Training.I: 5–18 on the BorgScale and 80% RM.F: 3 days/weekD: 60 - 100 minutes #S: 48 - 72	T0 = BaselineT1 = 6 monthsT2 = 18 months	ADAS-CogT0 = 8.02 (6.87 – 9.17)T1 = 6.26 (5.11 –7.41)T2 = 5.76 (4.59 – 6.92)	6 months intervention of a HIFT program improves global cognition compared to sham exercise; this benefit tend to persist for 18 months.
Gbiri et al., 2020 [36]	Dementia	CG= 15IG= 16	Basic HomeExercise Program.	69.6±3.4	Progressive Task-Oriented Circuit Training I: 80% RMF: 2 days/weekD: 70 minutes#S: 24	T0 = BaselineT1 = 6 weeksT2 = 12 weeks	MMSET0= 16.88T1= 3.56T2= 3.75	Progressive HIFT improves cognitive function.
Hoffmann et al., 2015 [37]	Dementia	CG= 88IG= 102	Usual care	CG 71.3±7.3IG 69.8±7.4	Moderate to high Intensity Aerobic exerciseI: 70 - 80% HRmaxF: 3 days/weekD: 60 minutes#S: 48	T0= BaselineT1= 16 weeks	MMSE T0= 23.8±3.4T1= 23.9±3.4	Exercise reduced neuropsychiatric symptoms in patients with mild Alzheimer disease, with possible additional benefits of preserved cognition in a subgroup of patients exercising with high attendance and intensity.
Lamb, et al., 2018 [38]	Dementia	CG= 165IG= 329	Usual care	CG 78.4±7.6 IG 76.9±7.9	Moderate to High Intensity Exercise Training I: 6-minute walk test for aerobic training and 20 RM for strength training. F: 2 days/weekD: 60 - 90 minutes#S: 30	T0 = BaselineT1 = 6 monthsT2 = 12 months	ADAS-CogT0= 21.2±9.5 T1= 22.9±11.6T2= 25.2±12.3	A four-month period of moderate to high intensity aerobic and strength exercise training, and ongoing support to exercise does not slow cognitive decline
Liu-Ambrose et al., 2016 [39]	MCI	CG= 35IG= 35	Usual care plus education	CG 73.7±8.3IG 74.8±8.4	Progressive Aerobic TrainingI: 40% to 70% of Heart Rate ReserveF: 3 days/weekD: 60 minutes#S: 78	T0= BaselineT1= 6 monthsT2= 12 months	ADAS-Cog T0= 11.7±5.5T1= -1.71 (-3.15 – -0.26)T2= -1.14±0.57	This study provides preliminary evidence for the efficacy of 6 months of thrice-weekly progressive aerobic training in community-dwelling adults with MCI, relative to usual care plus education.
Maffei et al., 2017 [40]	MCI	CG= 58IG= 55	Usual care	CG 74.9±4.4IG 74.0±4.8	Cognitive trainingF: 3 days/weekD: 120 minAerobic exerciseI: high intensity according to the ACSMF: 3 days/weekD: 60min	T0= BaselineT1= 7 months	ADAS-Cog T0= 9.92±4.81T1= -1.40±0.32	combined physical and cognitive training in a social setting improves cognitive status of MCI subjects and improves indicators of brain health.
Nascimento et al., 2014 [41]	MCI	CG= 17IG= 20	Usual care	CG 68.5±5.9 IG 67.3±5.3	I: 60 - 80% Heart rate reserveF: 3 days/weekD: 60 minutes#S: 48	T0= BaselineT1= 16 weeks	MoCAT0= 19 (4)T1= 23 (3)	a significant improvement of attention and executive functions in MCI group who took part in this exercise program was observed.
Rivas-Campo et al., 2023a [42]	MCI	CG= 68IG= 64	Usual care	CG 77.2±7.7IG 77.1±7.3	High Intensity Functional TrainingI: 80 - 85% HRmaxF: 3 days/weekD: 45 minutes#S: 36	T0= BaselineT1= 12 weeks	MoCA T0= 21.63±1.53T1= 22.58±1.41	After the analysis, improvement was found in the IG with significant differences with respect to the CG in the level of cognitive impairment (MoCA) (*p* < 0.001).
Sanders et al., 2020 [43]	Dementia	CG= 30 IG= 39	Flexibility exercises and recreational activities	CG 82,1±7,51 IG 81,7±7,16	Moderate to high intensity I= In the LI phase, the target RPE was 9–11. In the HI phase, the RPE was 13–16. F: 3 days/week D: 30 minutes #S: 72	T0=Baseline T1=12 weeks T2= 24 weeks	MMSE T0=21.4±3.94 T1=21.0±4.38 T2= 20.4±4.77	There were no significant effects of the exercise vs. control intervention on any of the cognitive measures.
Telenius et al., 2015a [44]	Dementia	CG= 79IG = 81	Light physicalactivity in sitting.	CG 86.4±7.8 IG 86.9±7.0	High Intensity Functional Exercise ProgramI: 12 RMF: 2 days/weekD: 50 - 60 minutes#S: 24	T0= BaselineT1= 12 weeks	MMSE T0= 15.6±5.0T1= 15.5±5.5	The results from our study indicate that a high intensity functional exercise program improved balance and muscle strength as well as reduced apathy in nursing home patients with dementia.
Telenius et al., 2015b [45]	Dementia	CG= 83IG= 87	Light physicalactivity in sitting.	CG 86.5±7.7 IG 87.3±7.0	High Intensity Functional Exercise ProgramI: 12 RMF: 2 days/weekD: 50 - 60 minutes#S: 24	T0= BaselineT1= 3 monthsT2= 6 months	MMSE T0= 15.5±0.6T1= 15.4 (14.5 – 16.3)T2= 14.4 (13.5 – 15.2)	The results demonstrate long-time positive effects of a high intensity functional exercise program on balance and indicate a positive effect on agitation
Toots et al., 2017 [46]	Dementia	CG= 93IG= 93	Activities in sitting.	CG 85.9±7.8 IG 84.4±6.2	High Intensity Functional Exercise ProgramI: 8 - 12 RMF: 2 days/weekD: 45 minutes#S: 40	T0=Baseline T1=4 monthsT2= 7 months	MMSET0= 15.4±3.4T1= -1.15±0.41T2= -2.25±0.42ADAS-CogT0= 31.8±11.4T1= 1.51±1.06	A 4-month, high-intensity functional exercise program had no superior effects on global cognition or executive function
Zhu et al., 2018 [47]	MCI	CG= 31IG= 29	Program of healthy lifestyle	CG 69.0±7.3 IG 70.3±6.7	DanceI: 60%–80% PHRsF: 3 days/weekD: 35 minutes#S: 39	T0= BaselineT1= 3 monthsT2= 6 months	MoCA T0= 23.2±1.9T1= 24.7±2.2T2= 25.0±2.4	The dance routine improves cognitive function, especially episodic memory and processing speed, In MCI patients

*CG* Control group, *IG* Intervention group, *LI *Low intensity, *HI* High intensity, *SCOG* Sham cognitive, *SPEX* Sham physical. *MMSE* Mini-Mental State Examination, *ADAS-Cog* Alzheimer’s disease Assessment Scale-cognitive, *MoCA* Montreal Cognitive Assessment, *D* Duration, *F* Frequency, *I* Intensity, *#S* Number of sessions, *HRmax* Maximum Heart Rate, *RM* Repetition maximum, *RPE* Rate of perceived exertion, *ACSM* American College of Sport Medicine, *MCI* Mild Cognitive Impairment

**Table 2:** Methodological quality of the included articles

Authorship	1	2	3	4	5	6	7	8	9	10	11	Total
Bossers et al., 2015 [34]	Y	Y	N	Y	N	N	Y	Y	N	Y	Y	6
Fiatarone et al., 2014 [35]	Y	Y	Y	Y	N	N	Y	Y	N	Y	Y	7
Gbiri et al., 2020 [36]	Y	Y	N	Y	N	N	Y	N	Y	Y	Y	6
Hoffmann et al., 2015 [37]	Y	Y	N	Y	N	N	Y	Y	Y	Y	Y	7
Lamb, et al., 2018 [38]	Y	Y	Y	Y	N	N	Y	Y	Y	Y	Y	8
Liu-Ambrose et al., 2016 [39]	Y	Y	Y	Y	N	N	Y	N	Y	Y	Y	7
Maffei et al., 2017 [40]	Y	Y	N	Y	N	N	Y	Y	Y	Y	Y	7
Nascimento et al., 2014 [41]	Y	Y	N	Y	N	N	Y	Y	N	Y	Y	6
Rivas-Campo et al., 2023a [42]	Y	Y	Y	Y	N	N	N	Y	N	Y	Y	7
Sanders et al., 2020 [43]	Y	Y	N	Y	N	N	Y	N	Y	Y	Y	6
Telenius et al., 2015a [44]	Y	Y	Y	Y	N	N	Y	Y	Y	Y	Y	8
Telenius et al., 2015b [45]	Y	Y	Y	Y	N	N	Y	Y	Y	Y	Y	8
Toots et al., 2017 [46]	Y	Y	Y	Y	N	N	Y	Y	Y	Y	Y	8
Zhu et al., 2018 [47]	Y	Y	Y	Y	N	N	Y	Y	N	Y	Y	7

Items: 1 = eligibility criteria; 2 = random allocation; 3 = concealed allocation; 4 = baseline comparability; 5 = blind subjects; 6 = blind therapists; 7 = blind assessors; 8 = adequate follow-up; 9 = intention-to-treat analysis; 10 = between-group comparisons; 11 = point estimates and variability; Y = Yes; N = No

### Methodological quality

The methodological quality of the included studies was assessed using the PEDro scale. Eleven studies were assessed using the PEDro website, while three studies [41, 42, 45] were evaluated manually. Overall, the 14 studies included in this review demonstrated a good methodological quality. However, it is important to note that none of the studies blinded participants or therapists (item 5 and 6). Additionally, six studies [34, 36, 37, 40, 41, 43] did not implement a concealed allocation of participants to groups (item 3) (Table 2).

### Global cognition

The main outcome in this review was global cognition. Several methods were employed to measure global cognition across the included studies. These methods included the Mini-Mental State Examination (MMSE) in studies [34, 36–38, 43–46], the Alzheimer’s Disease Assessment Scale-cognitive (ADAS-Cog) in studies [35, 39, 40], and the Montreal Cognitive Assessment (MoCA) in studies [41, 42, 47]. Among the included studies, seven [35, 36, 38–42] reported a statistically significant improvement in within-group analysis for participants who received the HIT intervention. Only one study [38] did not observe an improvement in cognition following HIT.

Additionally, although the remaining studies did not report significant differences, they did observe a deceleration in the progression of cognitive decline in the population that received the HIT intervention. These findings suggest a potential positive impact of HIT on cognitive function and a potential slowing of cognitive decline.

### Overall effect

**Fig. 2:**
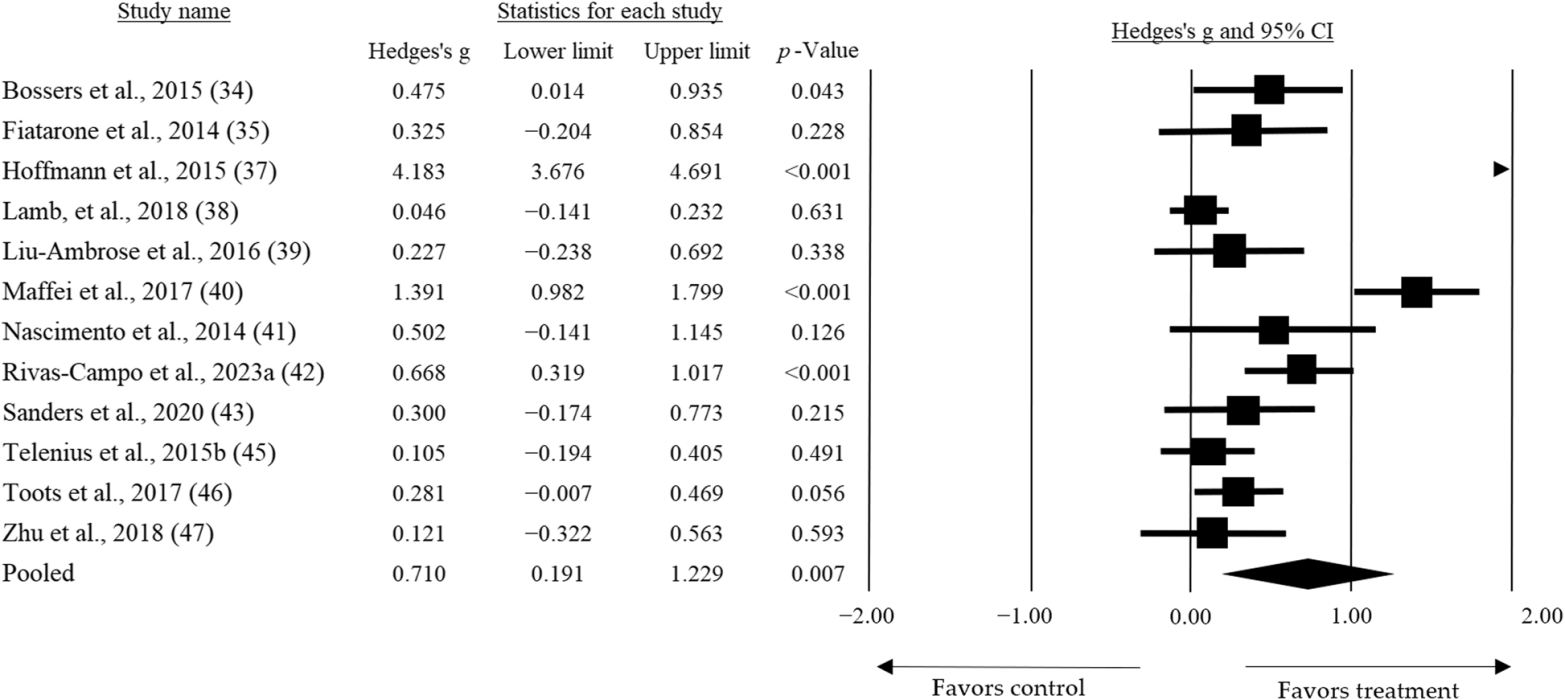
Forest plot of the overall mean effect size of high intensity exercise over global cognition

Out of the 14 articles included in this review, only 2 studies were not eligible for meta-analysis. One [36], was excluded due to the use of an intervention in the control group that differed from usual care or low-intensity exercise, while the other was excluded for duplicated data [44]. The meta-analysis considered a total of 998 older adults, with a mean age of 79.48 ± 6.42. Two models were considered for the meta-analysis: the fixed effects model and the random effects model. The random effects model was chosen to allow for the extrapolation of the results. Through the random effects model, it was revealed that HIT demonstrated a significant but small mean effect size of *g* = 0.710 (95% CI: 0.191 — 1.229; *p* = 0.007) on global cognition (Fig. 2).

### Dose-response

**Fig. 3:**
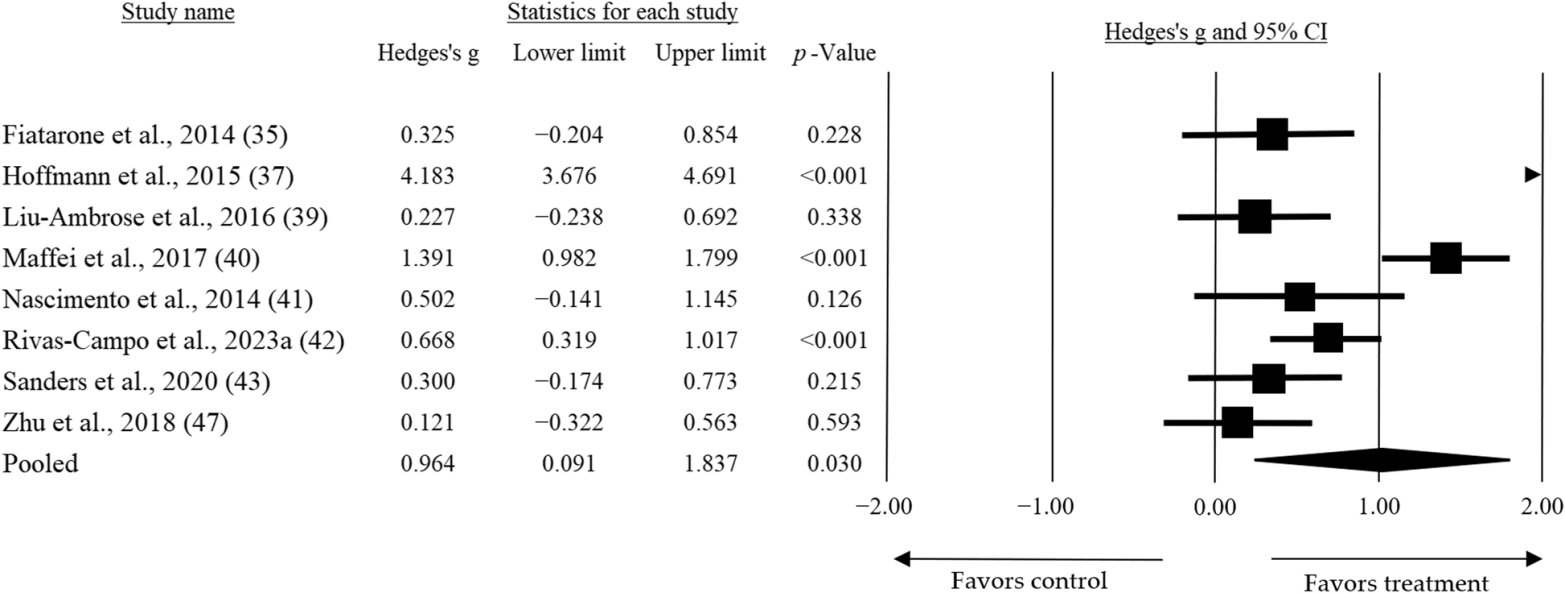
High-intensity training Mean effect size on global cognition practiced at low frequencies (3 times per week)

To determine the dose-response, a subgroup analysis was established, using frequency, time, and volume of interventions as moderating variables. In the case of frequency, the analysis revealed that the greatest mean effect size (*g* = 0.964, CI = 0.091 — 1.837, *p* = 0.030) was observed when interventions were performed three times per week (Fig. 3). On the other hand, three articles included interventions performed twice per week, while only one article performed the intervention four times per week, reporting a lower mean effect size. The effect size was not significant for the twice-per-week intervention (*g* = 0.113, CI = − 0.026 — 0.252, *p* = 0.110), whereas it was significant for the four-times-per-week intervention (*g* = 0.475, CI = 0.014 — 0.935, *p* = 0.043). Finally, considering the results of Hoffman et al. [37] as outliers, an analysis was performed excluding them. A smaller mean effect size was observed compared to when they were included, although it was still higher than the effect sizes obtained with other frequencies (*g* = 0.522, CI = 0.172 — 0.872, *p* = 0.003).

**Fig. 4:**
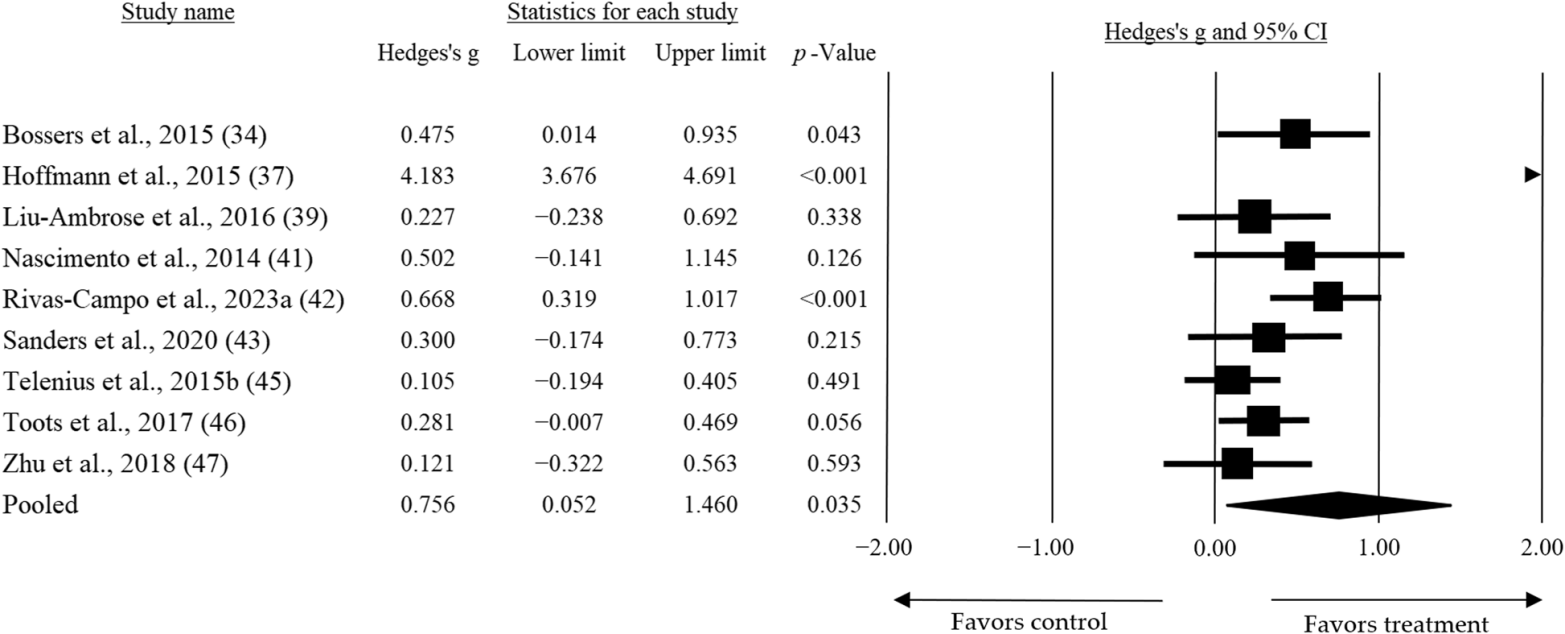
Mean effect size of High-intensity Training on global cognition practiced at short durations (≤ 60 min)

Regarding duration, the sub-group analysis allowed us to show that short-duration sessions (≤ 60 min) presented the greatest mean effect size (*g* = 0.756, CI = 0.052 — 1.460, *p* = 0.035) when compared to longer durations (> 60 min) (*g* = 0.585, CI = − 0.290 — 1.460, *p* = 0.190) (Fig. 4). Additionally, when considering the results of Hoffman et al. [37] as outliers, an analysis excluding their results showed a smaller but still significant mean effect size (*g* = 0.317, CI = 0.171 — 0.463, *p* < 0.001).

**Fig. 5:**
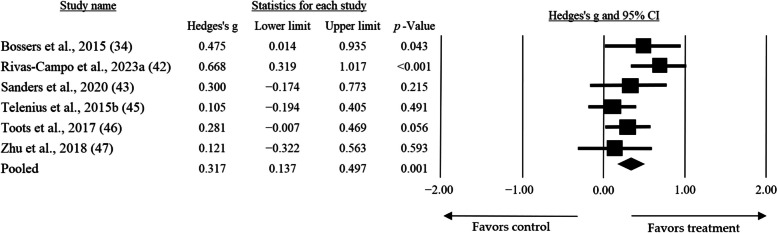
Mean effect size of High-intensity Training on global cognition when 135 min of work per week were completed

Finally, the weekly volume that presented a significant mean effect size was when the interventions reached ≤ 135 min per week of high-intensity work (0.317, CI = 0.137 — 0.497, *p* = 0.001). However, when the volume was higher, although the mean effect size increased, it was not significant (*g* = 1.109, CI = − 0.047 — 2.265, *p* = 0.060) (Fig. 5).

### Reporting biases

**Fig. 6:**
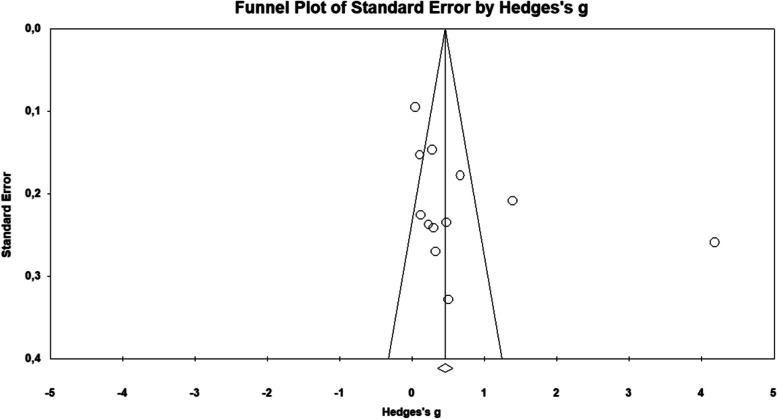
Funnel plot for publication bias

After analyzing the Funnel-plot graphically, it was possible to rule out a potential risk of publication bias due to the symmetry observed in the distribution of the graph (Fig. 6).

## Discussion

The objective of this systematic review with meta-analysis was to determine the optimal dose-response relationship of HIT for achieving maximum therapeutic effects in improving cognition in older adults with MCI or dementia. Out of the 14 studies included, only 1 [38] reported not having found favorable effects for the intervention, understood as improvements in global cognition or a decrease in the normal progression of cognitive impairment. Additionally, the meta-analysis determined that the best effects of HIT are obtained at low frequencies and durations, with sessions occurring three times per week and not exceeding 60 min individually. It is important to highlight that the volume that caused a statistically significant mean effect size was 135 min per week (0.317, CI = 0.137 — 0.497, *p* = 0.001).

Exercise, particularly at high intensities, generally has favorable effects on cognition, as demonstrated by our results and recent literature [49–51]. However, achieving improvement in cognition may not always be possible in older adults with cognitive impairment or dementia. This limitation arises from various subject-specific variables and their context, including dietary patterns, supplementation, sleep patterns, social engagement, and disease status [52]. Consequently, a delay in the natural progression of the pathology is regarded as a favorable outcome. Through meta-analysis, we determined that HIT has a positive, albeit medium, and statistically significant effect on global cognition (*g* = 0.710, 95% CI: 0.191 — 1.229; *p* = 0.007). This exercise intervention helps alleviate the decline in mental functions associated with MCI or dementia, in contrast to the control groups, which experienced a worsening of this variable in all included studies.

It has been estimated that approximately 23% of cases of MCI could be prevented if older adults engaged in vigorous physical activity at least three times per week [53]. This possibility arises from the multiple effects that HIT has on brain physiology and metabolism during the aging process. Furthermore, if exercise is performed throughout different stages of the lifespan, it accumulates positive effects and enhances the brain’s resilience to cognitive decline [54]. Emerging evidence suggests that metabolic changes in the brain, such as atypical protein aggregation, impairment of protein degradation pathways, disrupted axonal transport, mitochondrial dysfunction, and programmed cell death, play a role in the onset and progression of neurodegenerative disorders like MCI and Alzheimer’s disease [55]. In contrast, exercise has garnered substantial attention for its potential in mitigating cognitive decline, attributed to its beneficial effects on brain structure, neuroplasticity, and vascular function [56, 57]. However, it is essential to acknowledge that considerable heterogeneity exists among the exercise protocols employed in studies investigating its impact on cognition. This observation has been noted by Domingos et al. [8] in their systematic review with meta-analysis, as well as by other researchers [26, 27].

The heterogeneity observed in the intervention protocols proposed by the studies included in this systematic review with meta-analysis may account for the discrepant results reported by the authors. The meta-analysis indicated that low frequencies (three or less times per week) and short-duration sessions (approximately 60 min) are more effective in enhancing cognition in older adults with cognitive impairment or dementia. Previous research has already attributed favorable effects to exercise performed at low frequencies (one or two times per week) [58]. These findings align with the WHO’s “every step counts” recommendation and are consistent with the results observed by Gallardo-Gomez et al. [59], who reported clinically significant outcomes with exercise doses (frequency and duration) below the recommended levels.

Our systematic review and meta-analysis indicate that the relationship between HIT “dose”, understood as the result of the combination of frequency, duration, and volume, and cognitive function improvement is not straightforward. Specifically, higher doses do not necessarily lead to better outcomes. Instead, our findings suggest that optimal cognitive benefits are achieved with moderate doses, particularly when sessions are conducted three times per week and do not exceed 60 min each. This could be explained by adherence and fatigue related to exercise. Although there is no clear evidence on the modifiable factors and barriers to exercise adherence in older adults with MCI or dementia [60], it is logical to think that in shorter sessions, it is easier to maintain patients’ attention, thus sustaining the high intensity of the exercise.

This systematic review with meta-analysis possesses both strengths and limitations. On the positive side, the inclusion of studies with good or higher methodological quality according to the PEDro scale, the substantial number of participants across the studies, and the diverse range of countries where the studies were conducted enhance the generalizability of the results to various populations. However, there are some limitations to note. Firstly, when considering HIT, an adaptation period is typically required, which was not accounted for in this meta-analysis. Secondly, due to the observed heterogeneity, further studies that directly compare different exercise doses within two or more intervention groups are needed. Thirdly, it is important to acknowledge that for certain analyses, the number of available articles was limited, necessitating cautious interpretation of the results. Fourthly, the observed results apply only to older adults with MCI who do not have comorbidities that may affect their physical performance. Finally, although HIT is discussed, most of the included articles did not establish objective methods of measuring 1RM or HRmax. Therefore, new studies are necessary to verify the findings obtained by performing objective measurements.

## Conclusions

In conclusion, this systematic review with meta-analysis provides evidence that HIT at low doses, specifically 3 times per week with a duration of at least 60 min per session, yields clinically significant effects on global cognition in older adults with cognitive impairment or dementia. Nevertheless, it is crucial to interpret these findings with caution, considering the limited number of studies included in the meta-analysis and the observed high heterogeneity among them. Further research that directly compares the effects of different exercise doses is still required to enhance our understanding in this area.

## Data Availability

The datasets used and/or analyzed during the current study are available from the corresponding author on reasonable request.

## Abbreviations

MCI: Mild Cognitive Impairment
HIT: High Intensity Training
CI: Confidence Interval
IG: Intervention Group
CG: Control Group
MMSE: Mini-Mental State Examination
ADAS-Cog: Alzheimer's Disease Assessment Scale-cognitive
MoCA: Montreal Cognitive Assessment
LI: Low intensity
HI: High intensity
SCOG: Sham cognitive
SPEX: Sham physical
D: Duration
F: Frequency
I: Intensity
#S: Number of sessions
HRmax: Maximum Heart Rate
RM: Repetition maximum
RPE: Rate of perceived exertion
ACSM: American College of Sport Medicine
